# The Use of CRISPR/Cas9 Gene Editing to Confirm Congenic Contaminations in Host-Pathogen Interaction Studies

**DOI:** 10.3389/fcimb.2018.00087

**Published:** 2018-03-19

**Authors:** Jonathan Ferrand, Nathan P. Croft, Geneviève Pépin, Kerrilyn R. Diener, Di Wu, Niamh E. Mangan, John Pedersen, Mark A. Behlke, John D. Hayball, Anthony W. Purcell, Richard L. Ferrero, Michael P. Gantier

**Affiliations:** ^1^Centre for Innate Immunity and Infectious Diseases, Hudson Institute of Medical Research, Clayton, VIC, Australia; ^2^Department of Molecular and Translational Science, Monash University, Clayton, VIC, Australia; ^3^Department of Biochemistry and Molecular Biology, Infection and Immunity Program, Biomedicine Discovery Institute, Monash University, Clayton, VIC, Australia; ^4^Experimental Therapeutics Laboratory, School of Pharmacy and Medical Science, Sansom Institute for Health Research, University of South Australia, Adelaide, SA, Australia; ^5^Adelaide Medical School, Robinson Research Institute, The University of Adelaide, Adelaide, SA, Australia; ^6^Department of Periodontology, University of North Carolina at Chapel Hill, Chapel Hill, NC, United States; ^7^TissuPath Specialist Pathology, Mount Waverley, VIC, Australia; ^8^Integrated DNA Technologies Inc., Coralville, IA, United States; ^9^Department of Microbiology, Infection and Immunity Program, Biomedicine Discovery Institute, Monash University, Clayton, VIC, Australia

**Keywords:** *Salmonella*, CRISPR/CAS9, congenic mice, background contamination, host-pathogen interactions

## Abstract

Murine models of *Salmonella enterica* serovar Typhimurium infection are one of the commonest tools to study host-pathogen interactions during bacterial infections. Critically, the outcome of *S*. Typhimurium infection is impacted by the genetic background of the mouse strain used, with macrophages from C57BL/6 and BALB/c mice lacking the capacity to control intracellular bacterial replication. For this reason, the use of congenic strains, which mix the genetic backgrounds of naturally protected mouse strains with those of susceptible strains, has the capacity to significantly alter results and interpretation of *S*. Typhimurium infection studies. Here, we describe how macrophage knockout cell lines generated by CRISPR/Cas9 gene editing can help determine the contribution of background contaminations in the phenotypes of primary macrophages from congenic mice, on the outcome of *S*. Typhimurium infection studies. Our own experience illustrates how the CRISPR/Cas9 technology can be used to complement pre-existing knockout models, and shows that there is great merit in performing concurrent studies with both genetic models, to exclude unanticipated side-effects on host-pathogen interactions.

## Introduction

The interaction between a pathogen and the innate immune system, one of the first lines of host defense, is a crucial component in how an infection will resolve. *Salmonella enterica* serovar Typhimurium (*S*. Typhimurium) is a non-typhoidal *Salmonella* strain and is commonly used to study the role of innate immune pathways during host-pathogen interactions (Keestra-Gounder et al., 2015). *S*. Typhimurium infects the intestine, invades the bloodstream and disseminates to the mouse liver and spleen. Critically, the capacity of inbred mice to clear an *S*. Typhimurium infection is directly related to their genetic background (O'brien et al., 1980), with C3H/HeJ, C57BL/6, and BALB/c mice being more susceptible than CBA, 129 or A/J lines (Monack et al., 2004; Loomis et al., 2014; Keestra-Gounder et al., 2015). Susceptibility to acute *S*. Typhimurium infection partially depends on select genes at the interface of the host-pathogen interaction. For example, C57BL/6 and BALB/c mice bear a mutation in natural resistance-associated macrophage protein 1 (Nramp1) (Vidal et al., 1993), which is thought to control the concentration of cations required for intracellular bacterial survival (Goswami et al., 2001; Loomis et al., 2014). The effect of the C57BL/6 and BALB/c background on *S*. Typhimurium infection is not restricted to the mutation in *Nramp1*, however, as illustrated by the observation that transgenic C57BL/6 mice expressing functional Nramp1 still succumb to infection, which is not the case of 129 mice (Monack et al., 2004; Loomis et al., 2014).

Investigations of immune pathways involved in the response to *S*. Typhimurium infection often rely on genetically modified mice, where gene-specific targeting constructs alter gene expression. Originally, this was predominantly carried out using embryonic stem cells (ESCs) from the 129 mouse strain, implanted into blastocysts from C57BL/6 mice (Eisener-Dorman et al., 2009), resulting in chimera mice. Extensive backcrossing on the C57BL/6 background was then carried out to selectively enrich for the knockout alleles, and to reduce the 129 genetic material. At least 10 generations were considered necessary for removing 129 genetic contaminations, resulting in a theoretical 99.9% C57BL/6 genetic composition (Eisener-Dorman et al., 2009). Such mice are referred to as congenic mice, and are still widely used, with more than 8,000 lines from 129 ESCs recently cataloged (Vanden Berghe et al., 2015). Comparisons of the 129 and C57BL/6 genomes identified 1395 indels and single nucleotide polymorphisms in 1084 genes, leading to 188 gained/lost STOP codons (Vanden Berghe et al., 2015). Such widespread variations between 129 and C57BL/6 genomes mean that 99.5% of the 8,000 congenic lines analyzed were affected by ~20 passenger mutations within 10 centiMorgans of the gene targeted (Vanden Berghe et al., 2015). Consequently, although backcrossed >10 times, congenic mice are almost always affected by passenger mutations in flanking genes, originating from the 129 ESC-derived genetic material (Vanden Berghe et al., 2015). In addition, unlinked genetic loci from the 129 ESCs can also be randomly selected on the C57BL/6 background, in spite of the backcrosses (Eisener-Dorman et al., 2009).

Given the difference between C57BL/6 and 129 lineages in host- *S*. Typhimurium interactions, it is likely that certain phenotypes regarding *S*. Typhimurium infections in C57BL/6 congenic mice are wrongly attributed to a specific gene, rather than to genetic contamination from the 129 background. In this work, we originally observed a strong protective effect against *S*. Typhimurium infection in a *Tlr7*-deficient congenic line, and its associated primary macrophages. While attempting to uncover how *Tlr7-*deficiency conferred this protective effect, we discovered that these mice harbored a wild-type *Nramp1*^*G169*^ gene, normally mutated to *Nramp1*^*D169*^ in C57BL/6 mice. Given the strong effect of *Nramp1*^*G169D*^ mutation on *S*. Typhimurium bacterial replication *in vivo* and *in vitro* (Vidal et al., 1993; Govoni et al., 1999; Loomis et al., 2014), we speculated that the protective effects seen in *Tlr7*-deficient congenic mice and cells could be Nramp1-dependent, and mask a putative Tlr7 contribution. To gain further insight into the role of *Tlr7-*deficiency on *S*. Typhimurium infection, we repeated our studies in *Nramp1*^*G169D*^-mutant *Tlr7*-deficient macrophage cell lines generated with the CRISPR/Cas9 technology. These *in vitro* studies revealed that *Tlr7*-deficiency had no impact on *S*. Typhimurium infection. We propose that the concurrent use of CRISPR/Cas9 gene editing in macrophage cell lines and primary cells from pre-existing congenic knockout models should become standard practice in host-pathogen studies to exclude confounding results due to background contamination.

## Materials and methods

### Mice and ethics statement

The use of animals and experimental procedures were approved by the Monash Medical Centre Ethics Committee under reference MMCA/2010/53 and MMCA/2014/57. Colonies of C57BL/6J (ARC) and *Tlr7*^−/−^ mice (Wei et al., 2010) were maintained at the Reid Animal Facility, University of South Australia under specific pathogen-free conditions. For *Salmonella* Typhimurium infection, 8–12-week-old female mice were used.

### *Salmonella enterica* serovar typhimurium culture

*Salmonella enterica* serovar Typhimurium SL1344 were grown overnight in Luria Broth (LB) at 37°C and subcultured for an additional 3 h before being centrifuged, washed in PBS and resuspended in Dulbecco's modified Eagle's medium (DMEM). Bacterial suspensions were adjusted to 1 × 10^7^ colony forming unit (CFU)/ml after measurement of optical density at 600 nm.

### Animal infection and tissue processing

For oral infection, mice were infected by oral gavage with 100 μl of a suspension of *S*. Typhimurium at 1 × 10^7^ CFU/ml or with 100 μl LB, after sodium bicarbonate-neutralization of stomach acid. For intraperitoneal (i.p.) inoculation, 100 μl of a solution of *S*. Typhimurium at 1 × 10^4^ CFU/ml or 100 μl of LB was injected per mouse. Mice were culled by isoflurane overdose and cervical dislocation. Serum was collected in serum gel tubes and stored at −80°C until use. Half of the spleen and liver were homogenized in LB, and dilutions of homogenates were plated on LB agar plates for viable bacterial counts. Homogenates were spun down, and supernatants were complemented with an anti-protease cocktail before being used for ELISA. Half of the spleen and liver were fixed in 10% formalin and embedded in paraffin. Sections were stained with H&E and inflammatory scores were blindly assessed as follows. For the white pulp of the spleen: grade 0, normal; grade 1, reactive spleen; grade 2, coalescence; grade 3, microgranulomata; grade 4, loss of structure. For the red pulp of the spleen: grade 0, normal; grade 1, increase in neutrophil number; grade 2, microabscesses; grade 3, microabscesses and granulomata; grade 4, thrombosis and vasculitis. For the portal tract of the liver: grade 0, no inflammation; grade 1, acute or chronic inflammation confined to the portal tract; grade 2, acute or chronic inflammation expanding portal tract; grade 3, abscesses; grade 4, thrombosis and vasculitis. For the liver parenchyma, grade 0, no inflammation; grade 1, acute or chronic inflammation confined to the parenchyma; grade 2, acute or chronic inflammation expanding parenchyma; grade 3, abscesses; grade 4, thrombosis and vasculitis. Cumulative scores are shown (maximum of 8 per organ).

### Cell culture and stimulation

Primary bone marrow derived macrophages (BMMs) were prepared as previously described (Ferrand and Gantier, 2016). Briefly, femurs were flushed with DMEM complemented with 1x antibiotic/antimycotic and 10% fetal bovine serum (FBS) (referred as complete DMEM hereafter), and cells were plated in complete DMEM supplemented with 20% L-929 cell-conditioned medium in T75 flasks for 6 days at 37°C (with one change of medium on day 3). WT C57BL/6 macrophages BMMs were immortalized using the J2 retrovirus encoding v-raf and v-myc, as previously reported (Roberson and Walker, 1988). Briefly, primary BMMs differentiated in 20% L-929 cell-conditioned medium were infected with the retrovirus with Polybrene. 24 h after infection, the cells were rinsed and kept in 20% L-929 medium for 7 days. L-929 cell-conditioned medium was then withdrawn and the cells cultured in complete DMEM until stable growth was attained. RAW264.7 cells and immortalized WT C57BL/6 macrophages were also grown in complete DMEM. Cells were regularly tested for mycoplasma contaminations. TLR ligands were directly added to medium at the following final concentrations: 100 ng/ml Pam3CSK4 (TLR 1/2 agonist, InvivoGen), 10 μg/ml polyI:C (TLR 3 agonist, InvivoGen), 10 ng/ml LPS (TLR 4 agonist, LPS from E. coli Serotype O111:B4, Enzo Life Sciences), 1 μM R848 (TLR7 agonist, InvivoGen), 0.5–1 μM ODN1826 (TLR9 agonist, T^*^C^*^C^*^ A^*^T^*^G^*^ A^*^C^*^G^*^ T^*^T^*^C^*^ C^*^T^*^G^*^ A^*^C^*^G^*^ T^*^T, synthesized by IDT and resuspended in RNase-DNase free water) and 0.5 μg/ml Sa19 (TLR13 agonist, rG^*^rG^*^rA^*^ rC^*^rG^*^rG^*^ rA^*^rA^*^rA^*^ rG^*^rA^*^rC^*^ rC^*^rC^*^rC^*^ rG^*^rU^*^rG^*^ rG, synthesized by IDT and resuspended in RNase-DNase free TE buffer). “^*^” denotes a phosphorothioate modification. “r” denotes an RNA base.

### Gentamicin assay

On day 6 of differentiation, 1.5 × 10^5^ BMMs were seeded per well in a 24-well plates in complete DMEM with 20% L-929 cell-conditioned medium and incubated overnight at 37°C. Thirty minutes before infection, cells were washed twice with pure DMEM and incubated for 30 min before addition of 75 μl of a suspension of 1 × 10^7^ CFU/ml *S*. Typhimurium (MOI of 5). Infected cells were incubated for 45 min and rinsed with complete DMEM containing 100 μg/ml gentamicin for 10 min to remove extracellular bacteria. Cells were then incubated in complete DMEM containing 10 μg/ml gentamicin for an extra 5 h before lysis in 180 μl 0.2% Igepal CA-630 in PBS and plated on LB agar to determine viable bacterial counts arising from the intracellular contents. Supernatants were collected and stored at −80°C until cytokine levels were assessed by ELISA.

### mRNA isolation and RT-qPCR

Total RNA was purified from 1.5 × 10^5^ cells using the ISOLATE II RNA kit (Bioline) according to the manufacturer's instructions. cDNA was synthesized from isolated RNA using the High-Capacity cDNA Archive kit (ThermoFisher Scientific) according to the manufacturer's instructions. RT-qPCR was carried out with the Power SYBR Green PCR (ThermoFisher Scientific) on the HT7900 RT-PCR system (Life Technologies). Each polymerase chain reaction (PCR) was carried out in technical duplicate. Each amplicon was sequence-verified and used to generate a standard curve for the quantification of gene expression (used in each run). Melting curves were used in each run to confirm specificity of amplification. The primers used were the following: Mouse 18S rRNA: mRn18s-FWD GTAACCCGTTGAACCCCATT; mRn18s-REV CCATCCAATCGGTAGTAGCG and Mouse Arg1: mArg1-FWD CAGAAGAATGGAAGAGTCAG; mArg1-REV CAGATATGCAGGGAGTCACC.

### Microarray analyses

For microarray analyses, Cy3-labeled cRNA was prepared from 0.1 μg total RNA of BMMs infected 24 h with *S*. Typhimurium (MOI of 5), using the One-Color Low input Quick Amp labeling Kit (Agilent, v6.6 September 2012), followed by RNeasy column purification (Qiagen). 600 ng of Cy3-labeled cRNA was fragmented at 60°C for 30 min in 1x Agilent fragmentation buffer and 2x Agilent blocking agent. On completion, 25 μl of 2x Agilent gene expression hybridization buffer was added and 43 μl of sample hybridized for 17 h at 67°C in a rotating Agilent hybridization oven with one Agilent SurePrint G3 Mouse Gene Expression 8 × 60K (028005) microarray. After hybridization, microarrays were sequentially washed 1 min at room temperature with GE wash buffer 1 (Agilent) and 1 min with 37°C GE wash buffer 2 (Agilent). Slides were scanned on the Agilent C DNA microarray scanner using one-color scan settings for 8 × 60 k array slides. The scanned images were analyzed with Feature Extraction Software 11.0.1.1 (Agilent). Feature-extracted files are available in GEO (GSE103750), and were normalized and analyzed as follows. The probes that were not “Detected” in any of the 7 samples (from 3 wild-type and 4 *Tlr7*^−/−^) were removed. Log_2_ transformation and quantile normalization of the data were conducted. Array weights were computed using the “arrayWeights” function in the limma package. Correlated samples from 2 separate experiments were handled by random effect model through the R function “duplicateCorrelation” and were accommodated in the following linear model. We fitted the normalized data to a linear model using “lmFit,” while taking into account the array weights and batch sample correlation. Using the global FDR adjusted *P*-value of 0.05, 50 probes were up regulated in *Tlr7*^−/−^ and 41 down regulated in *Tlr7*^−/−^ comparing to WT cells with a log_2_ fold change ≥1. **Figure 2** is restricted to the genes with an official gene symbol. The log_2_ transformed quantile normalized data of the 7 samples were submitted to GEO (GSE103750). Normalized data for genes were processed with the Morpheus visualization tool (Broad Institute) for the creation of expression heat maps.

### CRISPR gene editing

Cas9 ribonucleoprotein (RNP) assembly was performed following the manufacturer's recommendations (IDT). Briefly, Alt-R® CRISPR-Cas9 crRNA and tracrRNA were annealed in equimolar concentrations to a final duplex concentration of 45 μM. The crRNA guide sequences employed were AUUGUGUACCUGUUCUACUGGUUUUAGAGCUAUGCU (for the *Tlr7*^−/−^ RAW264.7 clone #1 only) and UAUGGGACAUUAUAACAUCGGUUUUAGAGCUAUGCU (for all the other clones)—the *Tlr7*-specific protospacer element is underlined. 120 pmol of the resulting crRNA:tracrRNA was mixed with 100 pmol of recombinant Cas9 protein (Alt-R® Cas9 Nuclease 3NLS) and set to incubate at room temperature for 15 min. Electroporation transfection of 500,000 immortalized macrophages or RAW264.7 cells was performed using a mix of 90 μl of BTXPRESS electroporation buffer (BTX) and 10 μl of the Cas9 RNP complex in an AMAXA Nucleofactor II (program D032, Lonza). After 2 days recovery at 37°C, cloning by limiting dilution was performed and Tlr7 deficiency was identified by functional assay and gene sequencing. Control clones went through the same selection process. Sanger sequencing was used to confirm the genomic mutations, however one clone of *Tlr7*^−/−^ RAW264.7 cells (clone #1) failed to sequence (but was confirmed to be *Tlr7*-deficient by mass spectrometry).

### ELISA

IL-6 and TNF-α in culture supernatants or serum were detected using their respective BD OptEIA Mouse ELISA kits (BD Biosciences). Cxcl1 was detected in tissue homogenates using Mouse CXCL1 DuoSet ELISA (R&D systems). All kits were used in accordance with the manufacturer's instructions. Tetramethylbenzidine substrate (ThermoFisher Scientific) was used for quantification of the cytokines on a Fluostar OPTIMA (BMG Labtech) plate-reader.

### Protein digestion, LC-MS/MS and label-free quantitation

2.5 × 10^6^ BMMs were infected with *S*. Typhimurium as described in the gentamicin assay section (MOI 5). At 6 h post-infection, cells were washed in cold PBS before being harvested using a cell scraper in cold PBS. Cells were spun down, and pellets were snap-frozen in liquid nitrogen before being stored at −80°C prior to subsequent analysis. Cell pellets were lysed and processed by filter-aided sample preparation (FASP), as previously described (Wisniewski et al., 2009). Briefly, cells were lysed in 4% SDS, 0.1M Tris-HCl (pH 7.6), sonicated and then reduced with 5 mM *tris*(2-carboxyethyl)phosphine (TCEP) at 60°C for 30 min before being passed over a FASP column (Expedeon). Samples were alkylated and subjected to tryptic digestion on-column overnight at 37°C according to the manufacturer's instructions. Prior to mass spectrometry, FASP eluates were acidified and peptides were further purified and enriched using OMIX C18 Mini-Bed tips (Agilent Technologies). Using a Dionex UltiMate 3000 RSLCnano system equipped with a Dionex UltiMate 3000 RS autosampler, an Acclaim PepMap RSLC analytical column (75 μm × 50 cm, nanoViper, C18, 2 μm, 100Å; Thermo Scientific) and an Acclaim PepMap 100 trap column (100 μm × 2 cm, nanoViper, C18, 5 μm, 100Å; Thermo Scientific), tryptic peptides were separated by increasing concentrations of 80% ACN/0.1% formic acid at a flow of 250 nL/min for 95 min and analyzed with a QExactive Plus mass spectrometer (Thermo Scientific). Data was searched against the *Mus musculus* reference proteome (revision as of 2016-08), canonical and isoform sequences (consisting of 59,550 entries) using Maxquant (v1.5.2.8) (Cox and Mann, 2008; Cox et al., 2014) with the following settings: enzyme digestion set to trypsin (max missed cleavages of two); fixed carbamidomethyl cysteine modification; max variable modifications of five; minimum peptide length of 8 amino acids, maximum peptide length of 25 amino acids; known contaminants added to the search database; decoy database set to reverse; 1% false discovery rate (FDR) for peptide and protein identification; instrument parameters (including ppm tolerance settings) were the default settings for Orbitrap acquisition; label free quantitation (LFQ) was set to on and with a minimum ratio count of 2. For LFQ analysis, the Perseus (v1.5.3) software package was used (Tyanova et al., 2016). Raw LFQ values were Log_2_-transformed, reverse and contaminant sequences removed and rows set to require a minimum of three valid values across the six samples (three wild-type and three *Tlr7*^−/−^) (Supplementary Table 1). Any further missing values were replaced by imputation according to the normal distribution of the samples, with default values of width (0.3) and down shift (1.8) selected (Supplementary Table 2). Data were also analyzed by z-score normalization across each sample row (Supplementary Table 3). For a list of all identified peptides and assigned protein groups and MS metadata, refer to Supplementary Tables 4, 5. Raw MS data has been deposited on the ProteomeXchange PRIDE (PRoteomics IDEntifications database at the European Bioinformatics Institute) (Vizcaino et al., 2016) archive and can be accessed using the following accession number: PXD007239.

### Statistical analyses

Statistical analyses were carried out using Prism 6 (GraphPad Software Inc.). Two-tailed unpaired *t*-test or non-parametric Mann-Whitney *U*-tests were used to compare pairs of conditions. Symbols used: ns: not significant, ^*^P ≤ 0.05, ^**^P ≤ 0.01, ^***^P ≤ 0.001, ^****^P ≤ 0.0001.

## Results

### *Tlr7*-deficient mice are protected against *S*. typhimurium infection *in vivo*

In an observation that underlines the complexity of host-pathogen interactions, recruitment of the innate immune system does not always favor the host, and can also facilitate *S*. Typhimurium infection. This is illustrated with the case in interferon (IFN) receptor deficient (*Ifnar1*^−/−^) mice and *Nlrp12*-deficient mice that are partially protected against *S*. Typhimurium infection (Robinson et al., 2012; Zaki et al., 2014). In this study, we were interested in defining whether engagement of mouse Tlr7, which operates upstream of IFN production upon detection of endosomal RNA, could also play a role during *S*. Typhimurium infection. To this end, we infected *Tlr7*-deficient C57BL/6 mice with *S*. Typhimurium by oral gavage and initially assessed the survival of the mice compared to wild-type (WT) C57BL/6 mice. Importantly, the *Tlr7*^−/−^ colony used here was generated using the CJ7 129 ESC line (Lund et al., 2004), and had been backcrossed 10 times on the C57BL/6 background. Because Tlr-deficient lines are usually maintained homozygous, we used WT C57BL/6 from a different colony housed in the same room to ensure that both colonies were exposed to the same environmental entities (this is important for host-pathogen interactions).

**Figure 1:**
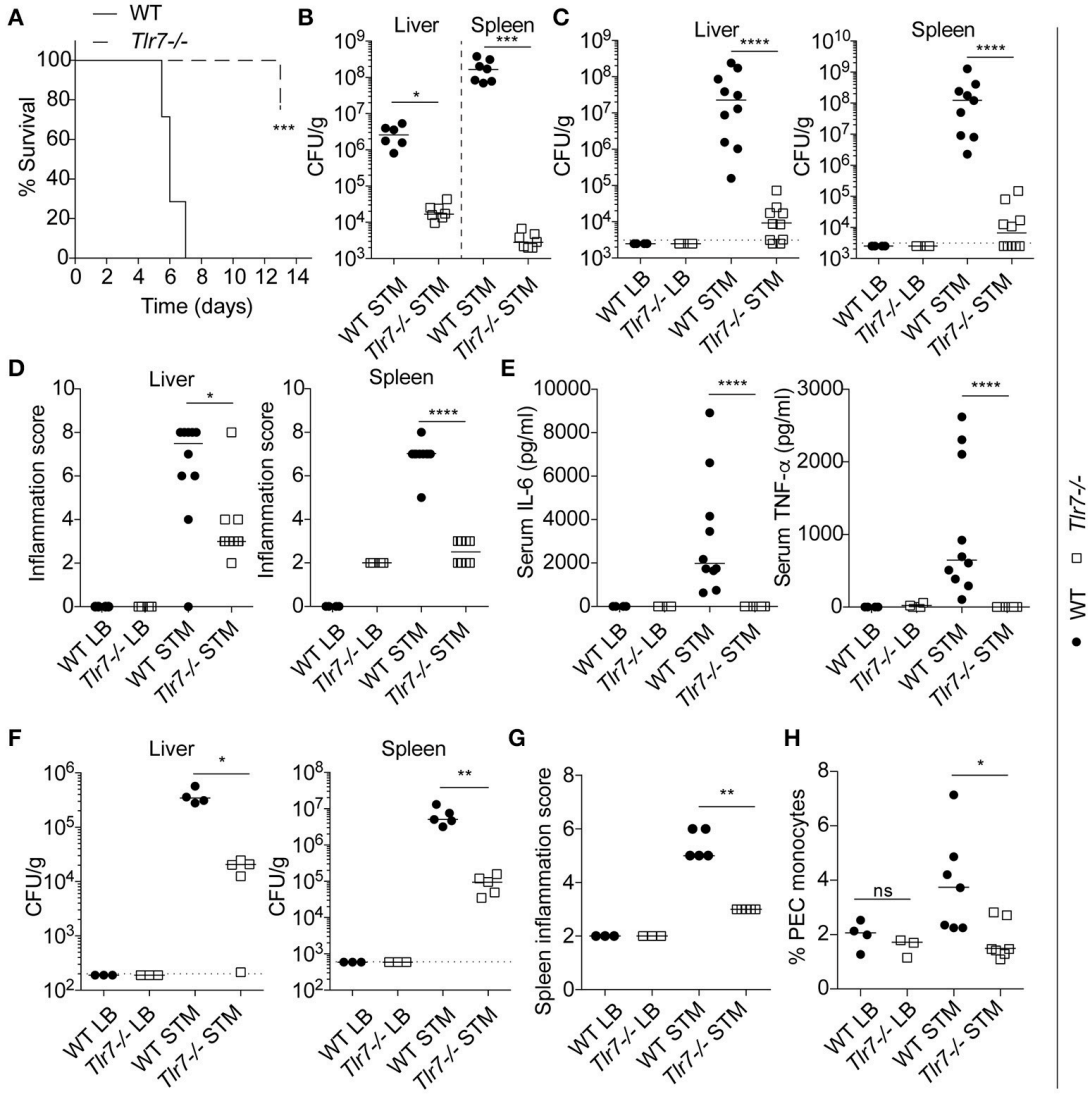
*Tlr7-*deficient mice are protected from *S*. Typhimurium infection *in vivo*. **(A–E)**. WT and *Tlr7*^−/−^ mice were infected with 1 × 10^6^ (oral route) CFU of *S*. Typhimurium. **(A)** Survival of infected mice was monitored for 13 days (*n* = 7 mice per group). STM: *S*. Typhimurium infected. Log-rank (Mantel-Cox) test comparing both genotypes is shown. **(B)** Bacterial count in liver and spleen was measured at time of death shown in **(A)** by CFU assay (*n* = 7 mice per group—a value for one WT mouse (liver) was under the detection limit and is not shown here). Median and unpaired Mann-Whitney *U*-tests are shown. **(C)** Bacterial counts in the liver and the spleen were measured at day 5 post-infection by CFU assay. LB: Luria Broth, i.e., non-infected. STM: *S*. Typhimurium infected. Data shown is averaged from two independent experiments (LB *n* = 3 mice; infected, *n* = 10 mice). Median and unpaired Mann-Whitney *U*-tests are shown. **(D)** Cumulative histological scores of liver and spleen from **(C)**. Median and unpaired Mann-Whitney *U*-tests are shown. **(E)** Serum levels of IL6 and TNF-α at day 5 post-infection were measured by ELISA from the mice used in **(C)**. Median and unpaired Mann-Whitney *U*-tests are shown. **(F,G)** WT and *Tlr7*^−/−^ mice were infected (intraperitoneal route) with 1 × 10^3^ CFU of *S*. Typhimurium. **(F)** Bacterial counts in the liver and the spleen were measured at day 3 post-infection by CFU assay (LB = 3 mice, STM infected = 5 mice—a value for one WT mouse (liver) was under the detection limit and is not shown here). Median and unpaired Mann-Whitney *U*-tests are shown. **(G)** Spleen cumulative histological score at day 3 post-infection from the mice used in **(F)**. Median and unpaired Mann-Whitney *U*-tests are shown. **(H)** Flow cytometry analysis of live monocytes (CD45^+^ CD11b^+^) from the peritoneal cavities at day 3 post-infection (oral route same as **A–E**). Data is averaged from two independent experiments (LB = 3 mice, infected = 7 mice) (median and unpaired Mann-Whitney *U*-tests are shown). **(C,F)** Dotted lines indicate the limit of detection. **(B,C,F)** CFU counts are shown relative to tissue weight. *P ≤ 0.05, **P ≤ 0.01, ***P ≤ 0.001, ****P ≤ 0.0001.

All the WT mice were highly sensitive to infection and reached humane endpoints by days 5–7, against only one *Tlr7*^−/−^ mouse by day 13, the day at which the experiment was stopped and all the *Tlr7*^−/−^ mice were culled (Figure 1A). Bacterial burdens in the liver and spleen were assessed at death, revealing strong protection in both organs of *Tlr7*^−/−^ mice, as reflected by >2 logs and >4 logs higher numbers of bacteria in the liver and spleen, respectively, of WT animals (Figure 1B). A similar observation was made when the mice from both genotypes were infected for a similar 5-day period (Figure 1C). Reduced bacterial replication in *Tlr7*^−/−^ mice was also associated with significantly decreased liver and spleen inflammation (Figure 1D), and strongly reduced serum pro-inflammatory IL-6 and TNF-α responses (Figure 1E). To define whether the protective effect of *Tlr7*-deficiency on *S*. Typhimurium infection was a function of the route of infection, we next infected the mice with *S*. Typhimurium via the intraperitoneal (i.p.) route. In agreement with our previous data, *Tlr7*^−/−^ mice were protected against i.p. infection, with significantly reduced bacterial load in the spleen and liver, and reduced inflammation at day 3 (Figures 1F,G). Finally, analysis of peritoneal exudate cells (PECs), and mesenteric lymph node (MLN) cells from both WT and *Tlr7*^−/−^ mice indicated that monocyte numbers were reduced in infected *Tlr7*^−/−^ mice (Figure 1H and not shown).

### *Tlr7*-deficient primary macrophages are protected against *S*. typhimurium infection *in vitro*

**Figure 2:**
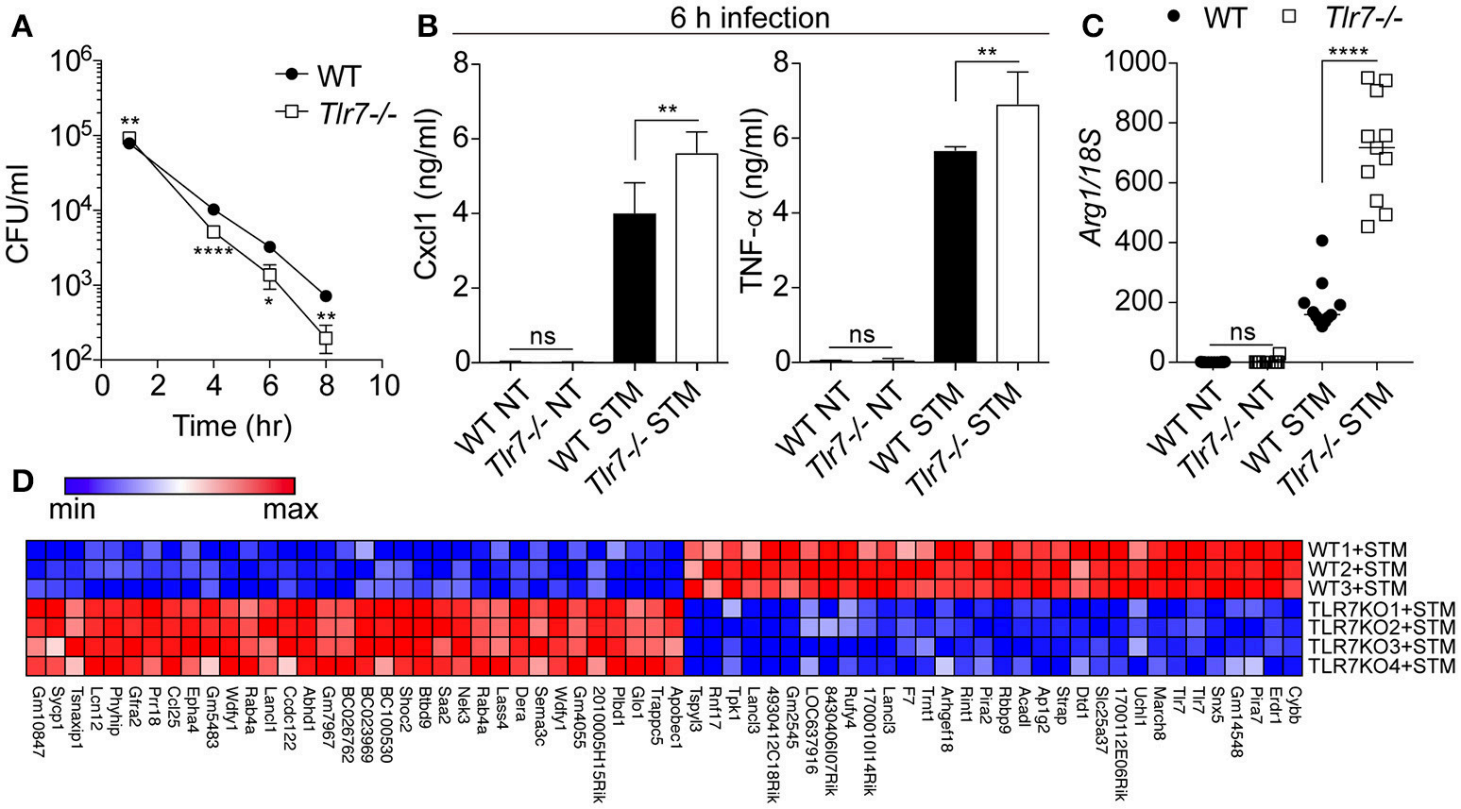
*Tlr7-*deficient BMMs clear *S*. Typhimurium more efficiently than WT BMMs. **(A)** Primary BMMs from WT and *Tlr7-*deficient mice were infected for indicated time prior intracellular survival assessment (gentamicin assay). NT: non-treated; STM: *S*. Typhimurium infected. Data shown is averaged from three mice in biological duplicate (± s.e.m and unpaired *t*-tests are shown), and is representative of 6 independent experiments. **(B)** Supernatants of cells from **(A)** were collected at 6 h and analyzed by specific ELISA. Data shown is averaged from three mice in biological duplicate (median with range and unpaired Mann-Whitney *U*-tests are shown), and is representative of 3 independent experiments. **(C)** RT-qPCR analyses of *Arg1* mRNA relative to 18S rRNA from BMMs infected for 24 h. Data shown is averaged from six mice in biological duplicate, from two independent experiments (median and unpaired Mann-Whitney *U*-tests are shown). **(D)** Microarray analysis of BMMs infected for 24 h with *S*. Typhimurium (each row represents BMM originating from a different mouse). TLR7KO refers to *Tlr7*^−/−^ cells. Only the significantly different genes are shown. *P ≤ 0.05, **P ≤ 0.01, ****P ≤ 0.0001.

Macrophages are an essential component of *S*. Typhimurium infection, being a cell type in which the bacterium can survive and replicate before disseminating to the spleen and liver (Keestra-Gounder et al., 2015). In light of the role of the monocytic compartment seen previously, we next assessed the role of Tlr7 in *S*. Typhimurium survival after infection of BMMs. In agreement with a protective role for *Tlr7*-deficiency against *S*. Typhimurium infection, we found that primary *Tlr7*-deficient BMMs were more efficient at inhibiting intracellular bacterial survival than their WT counterparts in the gentamicin assay (Figure 2A). This did not relate to decreased phagocytosis with *Tlr7*-deficiency given that bacterial survival was significantly greater in *Tlr7*-deficient BMMs after 1 h infection. Counter intuitively, *Tlr7*-deficient BMMs produced significantly more pro-inflammatory cytokines than WT cells, as evidenced by Cxcl1 and TNF-α levels (Figure 2B). We speculated that this heightened cytokine response *in vitro* could be due to the better capacity of the BMMs to kill the intracellular pathogen, alleviating some of its negative activities on innate immune responses. We hypothesized that macrophage polarization may have been impacted by the presence of Tlr7 and measured Arginase 1 (Arg1), a marker of macrophage phenotype, finding that expression was significantly increased upon infection in *Tlr7*-deficient BMMs (Figure 2C). To better understand the mechanisms at play in the protective action of *Tlr7-*deficiency, we next performed a microarray analysis of BMMs 24 h after infection with *S*. Typhimurium (Figure 2D). While WT and *Tlr7*-deficient BMMs exhibited different gene expression profiles, we were surprised to find that there was no enrichment for any pathways relating to immune function (“cysteine-type endopeptidase inhibitor activity” was the only GO term enriched) (Gene Ontology, 2015).

### Identification of a passenger mutation in *Nramp1* in *Tlr7*-deficient mice

**Figure 3:**
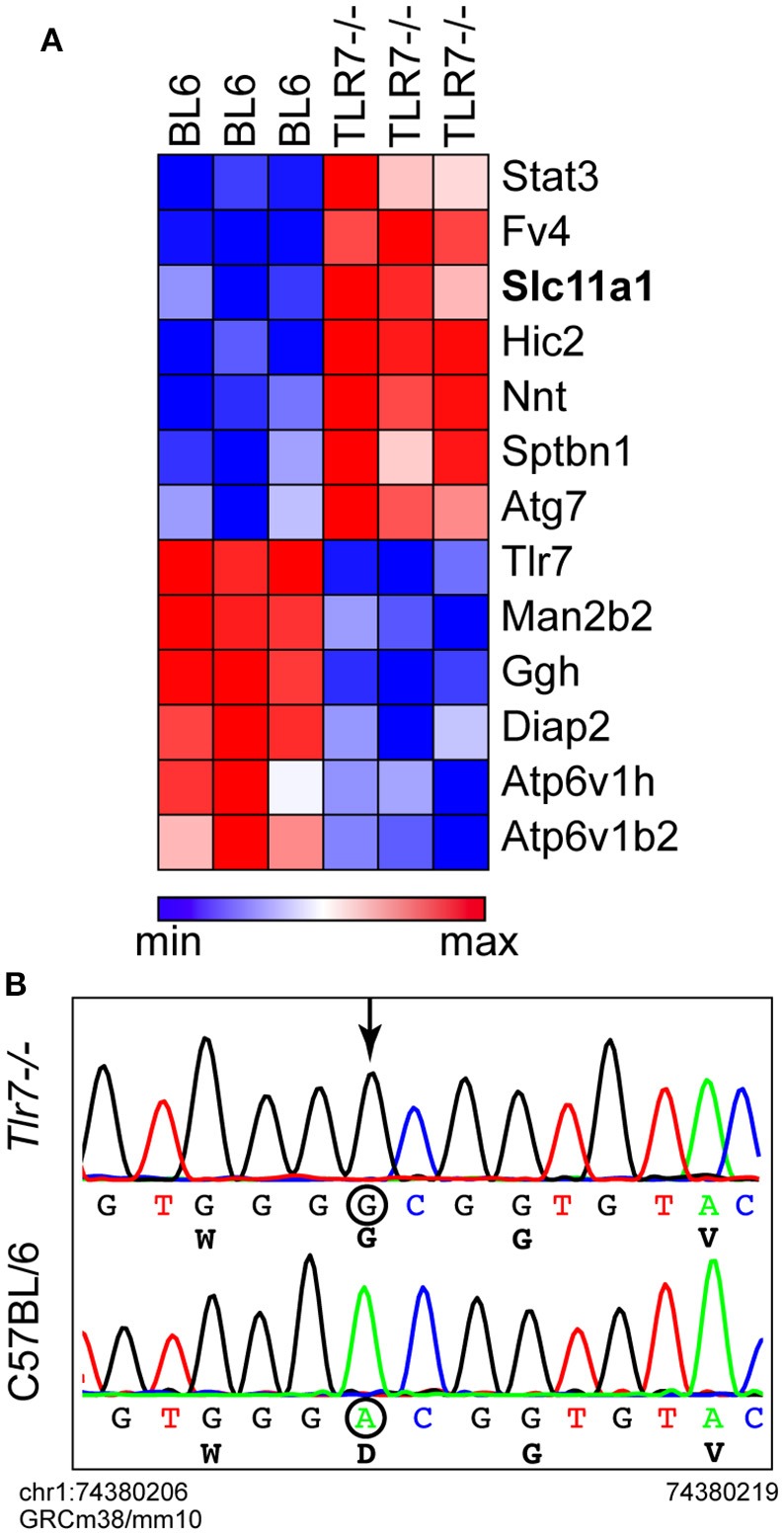
*Tlr7-*deficient mice have an *Nramp1*^*G169*^ allele. **(A)** Proteomic analysis of non-infected primary BMMs by LC-MS/MS (*n* = 3 mice). A small subset of differentially expressed proteins is shown here (normalized values used in this heatmap are provided in Supplementary Table 2). **(B)** Representative Sanger sequencing traces of the genomic region of *Nramp1*^*G169*^ from C57BL/6 WT and *Tlr7-*deficient mice. The localization of the sequence shown on chromosome 1 is provided for the first and last base shown (referenced to the GRCm38/mm10 genome).

To complement the microarray approach, we next resorted to mass spectrometry to analyze WT and *Tlr7*-deficient primary BMMs. Critically, when comparing the protein expression profile of non-infected primary BMMs, we noted that several proteins, in addition to Tlr7, were differentially expressed in WT and *Tlr7*^−/−^ BMMs (Figure 3A). While these basal differences could have been the result of an absence of Tlr7 itself or its downstream signaling (e.g., Stat3 or Fv4), we noticed that the gene encoding Nramp1 (*Slc11a1*), was expressed at much higher levels in *Tlr7*^−/−^ BMMs than their WT counterparts. C57BL/6 mice have an *Nramp1*^*G169D*^ mutation that is thought to destabilize the protein and prevent its normal expression (Vidal et al., 1996). Sanger sequencing of the *Nramp1* genomic region from the *Tlr7*-deficient mice confirmed that these mice expressed the wild-type *Nramp1*^*G169*^ allele of 129 origin, instead of the mutant *Nramp1*^*D169*^ allele normally found in WT C57BL/6 mice (Figure 3B). This finding suggested that the protective effect seen *in vivo* and *in vitro* in *Tlr7*-deficient mice and primary macrophages against *S*. Typhimurium infection, was presumably partially dependent on the *Nramp1*^*G169*^ allele—with similar protective effects of this allele previously reported *in vivo* and *in vitro* (Govoni et al., 1999; Loomis et al., 2014).

### *Tlr7^*CRISPR*−/−^* macrophage cell lines are not protected against *S*. typhimurium infection

**Figure 4:**
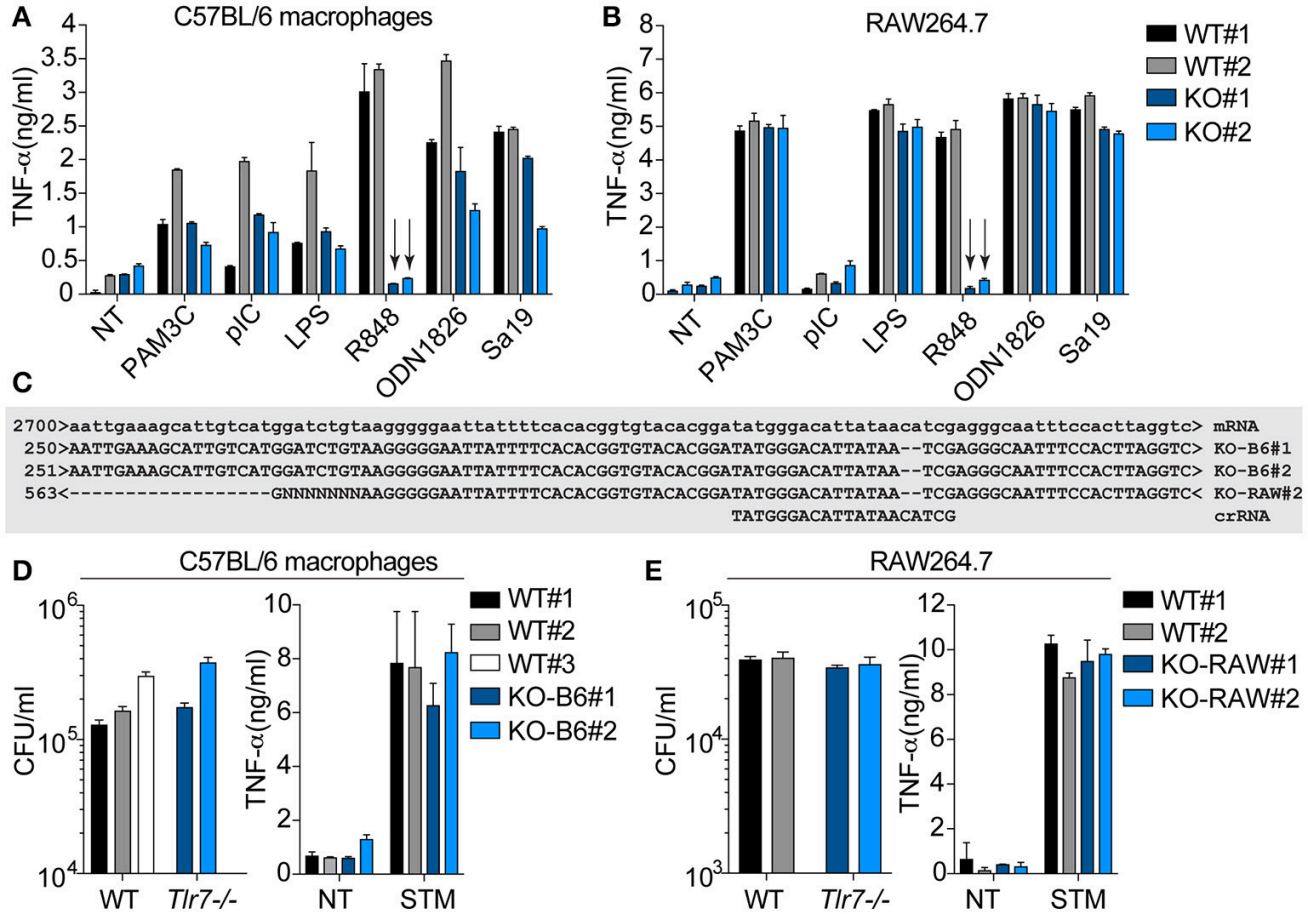
*Tlr7*^*CRISPR*−/−^ cells are not protected against *S*. Typhimurium infection. C57BL/6 immortalized macrophages **(A)** or RAW264.7 cells **(B)** electroporated with Tlr7-specific crRNA-tacrRNA and Cas9 (see Material and Methods section) were separated into WT and *Tlr7*-deficient clones (KO), after screening for their response to R848. Selected clones were further stimulated with indicated Tlr ligands: Pam3CSK4 (PAM3C) 100 ng/ml, polyI:C (pIC) 10 μg/ml, LPS 10 ng/ml, R848 1 μM, ODN1826 0.5–1 μM, Sa19 0.5 μg/ml). NT: non-treated. Supernatants were collected at 6 h and analyzed by TNF-α ELISA. Data shown is averaged from biological duplicate for each clone studied, and representative of a minimum of 3 independent experiments for LPS and R848 (± s.e.m shown). The arrows point to the loss of Tlr7 response to R848 in the selected clones. **(C)** Sequence alignment of *Tlr7*-deficient clones obtained from Sanger sequencing of purified genomic DNA. The *Tlr7*-specific protospacer element in the crRNA is shown. The clone KO-RAW#1 did not amplify by PCR, but was confirmed to be *Tlr7*-deficient by mass spectrometry. Intracellular survival of *S*. Typhimurium in C57BL/6 immortalized macrophage clones **(D)** or RAW264.7 clones **(E)** was assessed by gentamicin assay [in biological triplicate **(D)** or quadruplicate **(E)**], at 6 h post-infection. Supernatants were also collected at this time point and analyzed for TNF-α levels. The survival data shown is representative of 3 independent experiments **(D)**. NT: non-treated; STM: *S*. Typhimurium infected.

To delineate the putative role of Tlr7 in *S*. Typhimurium infection, independent of *Nramp1*^*G169*^, we generated two sets of WT and *Tlr7*-deficient clones, with the transient transfection of synthetic guide RNAs together with recombinant Cas9 into RAW264.7 macrophages (derived from a BALB/c mouse), or into immortalized BMMs (derived from a C57BL/6 mouse)—both cell models being based on the mutant *Nramp1*^*D169*^ allele. It is noteworthy that RAW264.7 cells are an established model of *S*. Typhimurium infection, in which the protective effect conferred by a rescue of the *Nramp1*^*G169*^ allele has previously been demonstrated (Govoni et al., 1999). Selective Tlr7 functional ablation was confirmed using the synthetic ligand R848, and the function of other TLRs was unaltered (Figures 4A,B). The *Tlr7*-deficient cells were also validated by Sanger sequencing (Figure 4C) or mass spectrometry (not shown). Analysis of *S*. Typhimurium intracellular survival after phagocytosis by these cell lines did not show any significant difference in the absence of Tlr7 expression (Figures 4D,E). In line with this, the *Tlr7*^*CRISPR*−/−^ cells failed to produce more pro-inflammatory cytokines than the WT cells upon *S*. Typhimurium infection (Figures 4D,E). These findings collectively supported that *Tlr7*-deficiency alone did not have any significant impact on *S*. Typhimurium infection in immortalized macrophages, *in vitro*, suggesting further that our observations in primary BMMs from *Tlr7-*deficient mice were independent of Tlr7.

## Discussion

Background genetic contamination in animal studies has been known for about 20 years, but has been often ignored by researchers (Vanden Berghe et al., 2015). In 2011, however, the discovery of a key role for a *Casp11* mutation of 129 origin in a phenotype previously attributed to its neighboring gene *Casp1*, reignited interest in the importance of ESC-derived genetic material in the phenotype of congenic mice (Kayagaki et al., 2011). Following on from this work, a recent study indicates that up to 80% of genetically modified mice cataloged in the “Mouse Genome Informatics” database were generated through the use of ESCs from the 129 strain, in which nearly all target genes were potentially accompanied by mutations in neighboring genes affecting their expression or function (Vanden Berghe et al., 2015). In light of these contaminations and the differences in host-pathogen interactions between 129 and C57BL/6 mice, it is critical to control for unintended background mutations when conducting host-pathogen studies.

One simple way to minimize the impact of such background contaminations is to rely on the use of littermate controls from heterozygote breeders rather than WT controls (Holmdahl and Malissen, 2012). In our study, we compared *Tlr7*-deficient congenic mice to non-littermate WT control mice on a C57BL/6 background, which is common practice in the field of innate immunity to facilitate the comparison of several knockout lines and to reduce the cost of heterozygous breeding. While originally attributing the strong protective phenotype of *Tlr7*-deficiency against *S*. Typhimurium infection to Tlr7, mass spectrometry analyses revealed that our *Tlr7*-deficient congenic mice harbored a wild-type allele of *Nramp1* (*Nramp1*^*G169*^), which is normally mutated into *Nramp1*^*D169*^ in C57BL/6 mice (Vidal et al., 1993, 1996). The similarity of protection observed in our *in vivo* experiments with those reported by others in *Nramp1*^*G169*^ congenic animals (Loomis et al., 2014), suggested that at least some of the phenotypes observed in our *Tlr7*-deficient congenic mice (i.e., decreased bacterial load in liver and spleen, and intracellular survival in primary BMMs), were in fact due to the presence of *Nramp1*^*G169*^ in these animals. In agreement with this, there was no significant impact of *Tlr7*-deletion by CRISPR/Cas9 in *Nramp1*^*D169*^ macrophage cell lines on *S*. Typhimurium infection. Although these *in vitro* observations do not rule a possible contribution of *Tlr7*-deficiency to the protective phenotype seen *in vivo*, independent of *Nramp1*^*G169*^ and other putative passenger mutations present in our line, they suggest that the role of Tlr7 is likely to be limited, given that the macrophage compartment is essential in the control of infection (Keestra-Gounder et al., 2015). Nonetheless, further *in vivo* infection studies in another colony of C57BL/6 or BALB/c *Tlr7*-deficient mice will be necessary to firmly rule out a role of Tlr7 on *S*. Typhimurium infection.

Our own journey illustrates the necessity for studies relying on congenic strains to use littermate controls to minimize the contributions of background mutations in phenotype interpretations. While littermate breeding would most likely have avoided the initial interpretation of a protective effect of *Tlr7*-deficiency, it would not have prevented the carryover of putative flanking mutations from the 129 strain. Ideally, isogenic lines should be preferred to congenic lines, but in light of their near ubiquity, it is clear that many researchers will keep on using congenic lines for some time. Importantly, it was recently suggested that Cre/*loxP*-based conditional gene deletion would avoid most passenger mutations, and as such, may constitute a better alternative, when available, to studies with congenic mice (Vanden Berghe et al., 2015). Nevertheless, such approaches have their own limits and have the potential to introduce “Cre effects,” possibly instigating potent immune responses (Pepin et al., 2016).

Before discovering the *Nramp1*^*G169*^ allele in our congenic animals, our data was converging toward a key role for Tlr7 in the control of *S*. Typhimurium infection. While unfortunate, our experience is unlikely to be unique. It is most probable that other works relating to host-pathogen interactions have wrongly attributed an effect to a knockout gene, when in fact pertaining to a congenic contamination. To prevent this, and beyond a systematic genomic sequencing analysis of each strains studied, we propose here that phenotypic validation of host-pathogen interactions from congenic lines can be rapidly carried out using immortalized cell lines generated with CRISPR/Cas9 gene editing. CRISPR/Cas9 allows for the rapid generation of immortalized cell lines with gene knockout activity through recruitment of the Cas9 enzyme to a target locus by a specific guide RNA, and the ensuing non-homologous end joining repair that can ablate protein translation (Jinek et al., 2012, 2013; Cong et al., 2013). In our study, we relied on a transient CRISPR/Cas9 activity, through the use of an electroporated synthetic guide RNA complex and recombinant Cas9 in mouse macrophage lines. Screening of *Tlr7*^*CRISPR*−/−^ clones was performed through functional inhibition of Tlr7 sensing of its chemical ligand, and indels were confirmed by Sanger sequencing. This approach was relatively rapid (~4 weeks), and inexpensive. Given the importance of macrophages in *S*. Typhimurium infection, this approach is very pertinent to this pathogen, and should therefore become standard practice to complement data from primary cells from congenic mice, to help clear potential background contaminations existing in the primary cells.

Finally, it is clear that CRISPR/Cas9 gene editing has its own caveats, as illustrated with a recent genome sequencing analysis of CRISPR/Cas9-mice that revealed the off-target mutation of >40 gene exons (Schaefer et al., 2017). With the increasing popularity of CRISPR/Cas9-mice for the study of gene function, one should probably also consider how to examine and account for unintended targeting in the interpretation of host-pathogen interactions. Given the ever expanding tool box available to study gene function, we suggest that when applicable *in vitro* models are available (as seen here with immortalized macrophages and *S*. Typhimurium), another gene targeting approach should be used to validate the phenotype of primary cells derived from CRISPR/Cas9-mice, during host-pathogen interactions. Whether it is with stable small hairpin RNA expression, zinc-finger nucleases, TALENs or congenic cell lines, the concurrent use of two independent genetic models should really become standard practice in the field, to alleviate the unanticipated effects of the genetic model itself.

## Author contributions

JF designed, performed, analyzed experiments and helped draft initial versions of the manuscript. NC performed proteomics experiments and analyses. KD helped maintain the *Tlr7*-deficient colony and helped analyze experiments. DW performed microarray bioinformatics analyses. NM helped with acquisition and interpretation of FACS analyses of *Tlr7*-deficient mice. JP performed blinded scoring of tissue inflammation. GP helped with acquisition and analysis of serum cytokines. MB helped with the design and synthesis of CRISPR/Cas9 reagents. JH assisted in the design and analysis of animal studies. AP assisted in the analysis of proteomics experiments. RF assisted with the design of experiments. MG conceived and coordinated the study, assisted in design and analysis of experiments, and drafted the manuscript. All authors reviewed the results and approved the final version of the manuscript.

### Conflict of interest statement

MB is employed by Integrated DNA Technologies, Inc., (IDT) which offers reagents for sale similar to some of the compounds described in the manuscript. IDT is, however, not a publicly traded company and the author does not personally own any shares/equity in IDT. The other authors declare that the research was conducted in the absence of any commercial or financial relationships that could be construed as a potential conflict of interest.
